# 3D dataset of a twisted bending-active beam element digitized using structure-from-motion photogrammetry

**DOI:** 10.1016/j.dib.2024.110254

**Published:** 2024-02-28

**Authors:** Mateusz Janiszewski, Serenay Elmas, Athanasios A. Markou, Joonas Jaaranen, Günther H. Filz

**Affiliations:** a Department of Civil Engineering, School of Engineering, Aalto University, Finland; b Department of Architecture, School of Arts, Aalto University, Finland; c Lightweight Structures Unit (LSU), Faculty of Architecture, Universität Innsbruck, Austria

**Keywords:** Digitization, 3D reconstruction, Plywood, Bending-active, Large deformation, Data processing

## Abstract

The current work presents the generation of a comprehensive spatial dataset of a lightweight beam element composed of four twisted plywood strips, achieved through the application of Structure-from-Motion (SfM) - Multi-view Stereo (MVS) photogrammetry techniques in controlled laboratory conditions. The data collection process was meticulously conducted to ensure accuracy and precision, employing scale bars of varying lengths. The captured images were then processed using photogrammetric software, leading to the creation of point clouds, meshes, and texture files. These data files represent the 3D model of the beam at different mesh sizes (raw, high-poly, medium-poly, and low-poly), adding a high level of detail to the 3D visualization. The dataset holds significant reuse potential and offers essential resources for further studies in numerical modeling, simulations of complex structures, and training machine learning algorithms. This data can also serve as validation sets for emerging photogrammetry methods and form-finding techniques, especially ones involving large deformations and geometric nonlinearities, particularly within the structural engineering field.

Specifications Table

**Table:** 

Subject:	Civil and Structural Engineering, Computer Graphics and Computer-Aided Design
Specific subject area:	Using Structure-from-Motion and Multi-view Stereo photogrammetry for 3D digitization of a lightweight beam comprising four twisted plywood strips.
Data format:	• Point cloud (.xyz) • Meshes (.obj) with associated material settings file (.mtl) and textures (.png) • Images (.jpeg)
Type of data:	• High-resolution 3D point cloud • 3D meshes in three different sizes: high-poly, medium-poly, and low-poly, with the associated texture files (6 color maps for high-, medium-, and low-poly meshes, and 6 normal maps for medium-, and low-poly meshes) • 122 images for photogrammetric reconstruction of the 3D model
Data collection:	Data were acquired through Structure-from-Motion (SfM) Multi-view Stereo (MVS) photogrammetry, employing a Canon EOS 5DS R camera with a Canon 35 mm F1.4L II lens, set to ISO 100 and f/11 aperture. A total of 122 images (8688 × 5792 pixels each) with 70 % overlap were taken. For scaling and control, six scale bars were utilized. The software used was RealityCapture 1.2.1.116300. The alignment was set on high mode, leading to 122 images aligned. The reconstruction of the 3D models was done in normal mode, resulting in high-poly, medium-poly, and low-poly meshes that were textured to ensure the highest quality results for further analysis.
Data source location:	• Department of Civil Engineering, School of Engineering, Aalto University • Espoo • Finland • 60°11′14.1"N 24°49′54.8"E
Data accessibility:	Repository name: Mendeley Data [1]. Data identification number: doi:10.17632/4yy65wd7y6.2 Direct URL to data: https://data.mendeley.com/datasets/4yy65wd7y6/2
Related research article:	Elmas, S., Jaaranen, J., Markou, A. A., Filz, G. H., & Koponen, S. [2]. Geometrically nonlinear behaviour of actively twisted and bent plywood. Engineering Structures, 302,117300. 10.1016/j.engstruct.2023.117300

## 1 Value of the Data

• The high resolution of the data allows for precise understanding and analysis of the beamʼs geometry, as a result of large deformations. Provided data set can be used for validation and calibration of the digital simulations (numerical) in the field of bending-active structures, which is necessary for their further formal and structural investigations in digital environment.
• Although there is a need for validation of numerical simulations, where the thin flexible strips undergo large deformations, there is a lack of available digitized data obtained from physical prototypes to inform the numerical simulations.
• Researchers specializing in bending-active structures, which have gained significant attention over the past decade, along with software and plug-in developers, can utilize the presented data.
• Validating the numerical models can establish a baseline for designing similar structures, providing an opportunity to conduct comparative studies, for example using data as a benchmark case.
• These data could be incorporated into machine learning models aimed at recognizing or designing similar structures, providing a rich resource for training such models.

## 2 Background

**Fig. 1:**
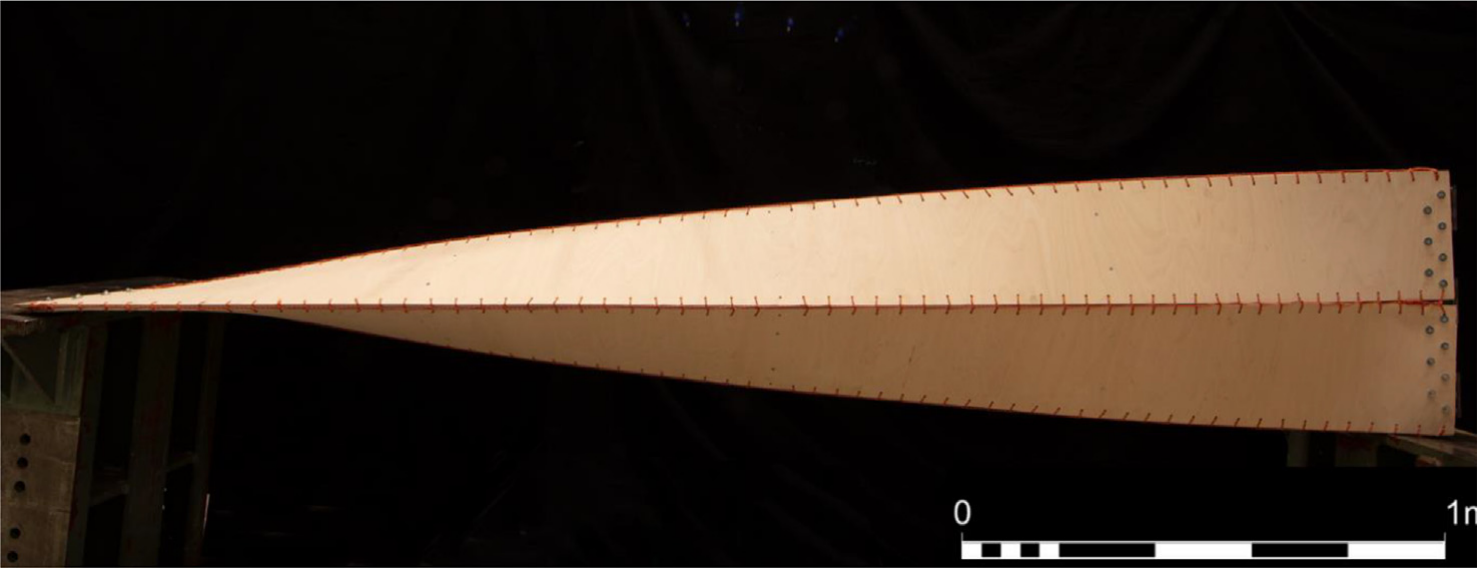
Side view of the full-scale beam element, composed of four twisted plywood strips.

The dataset was generated to create a 3D reconstruction of a beam element composed of four thin, twisted plywood strips, which has been employed in two different architectural applications, see Fig. 1. To form the beam element, each identical strip deforms under a 90o twist coupled with bending in two different directions, as explained in [2]. Since the proposed formation process of the beam element involves large deformations, including geometric non-linearities, its numerical modeling is challenging. Thus, validating and calibrating against real prototypes is essential to ensure the reliability of digital simulations and further explorations, such as, stress analysis.

To this end, high-quality images were captured and processed utilizing advanced Structure-from-Motion (SfM) and Multi-view Stereo (MVS) photogrammetry techniques to produce to generate the digital model. This data article provides an in-depth overview at the comprehensive methods used to gather and process the data. By providing access to the raw data, mesh, and texture files, other researchers and professionals can utilize our methodology, replicate the results, or base their related studies on these data. This transparency encourages continued innovation in the field of lightweight structures, specifically in the area of numerical modeling of the thin plates that involved large deformations.

## 3 Data Description

**Fig. 2:**
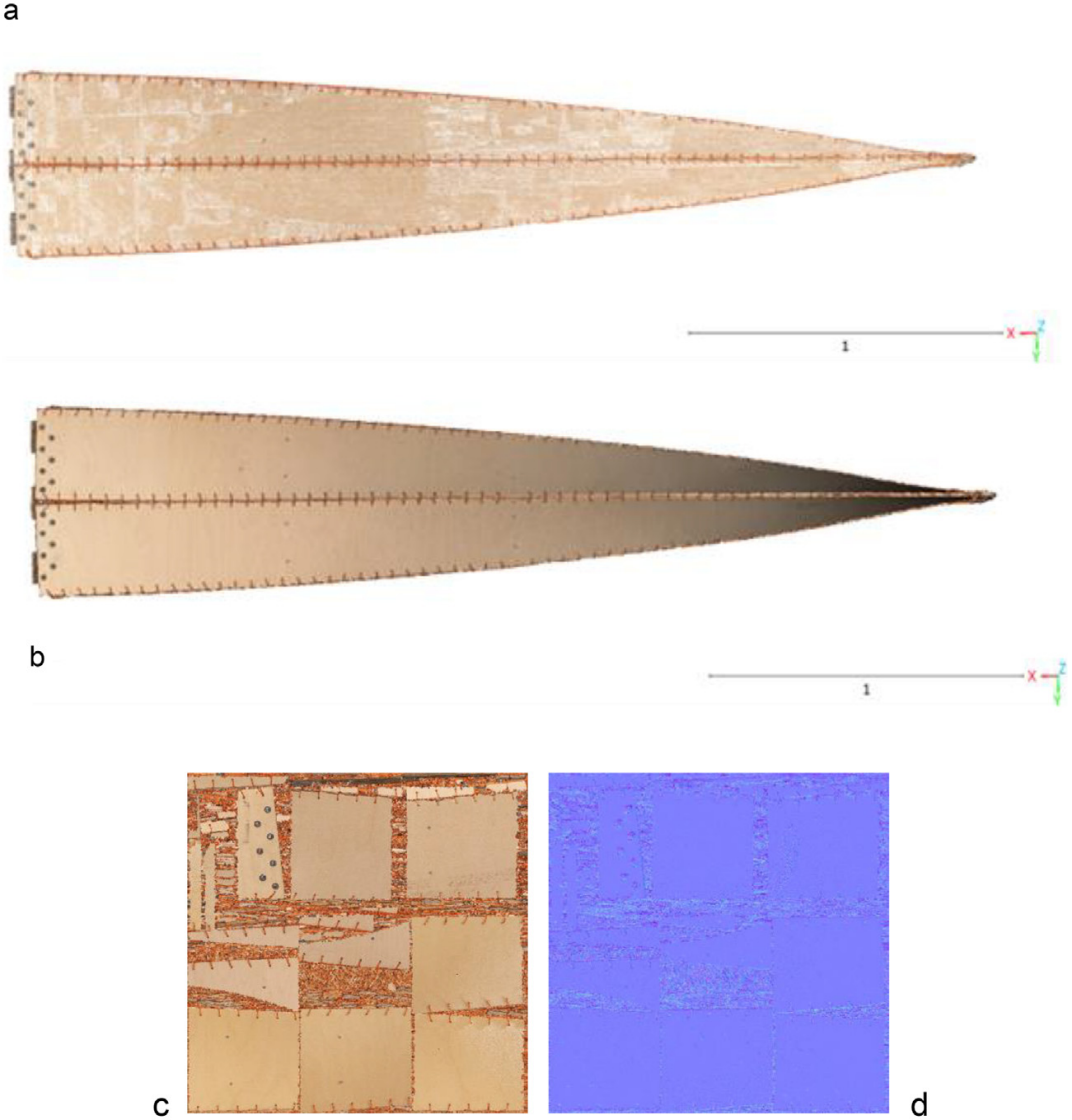
Illustrates the three different data files that can be find in the repository: a point cloud file (a), a mesh file (b), and textures files (c,d).

The data shared in this article comprise three raw data files: a point cloud file, a mesh file, and textures file, see Fig. 2.

Point Cloud File: This file, saved as a .xyz format, contains a collection of colored data points acquired from the photogrammetry process. Each data point in this file represents a point in a three-dimensional coordinate system, capturing the 'shape' of the object or space being imaged with a XYZ coordinates, NxNyNz normals, and a RGB value for each point representing its color.

Mesh File: Stored in the .obj format, this file is an integral part of the 3D modeling process. It contains a set of polygons that form the graphical representation of the structure or object captured in the photogrammetry process. The mesh is created by connecting the data points from the point cloud file to create a 3D object. Each mesh has six texture files associated with it, which are described below.

Textures File: This file, saved in .png format, comprises the textures applied to the 3D model created from the high-resolution images. It provides visual details (color maps and normal maps) that enhance the realism of the 3D model.

It is important to note that digitized model represents the aforementioned built beam element and not an ideal shape.

## 4 Experimental Design, Materials and Methods

The primary methodology used in this project was Structure-from-Motion (SfM) Multi-view Stereo (MVS) photogrammetry, a mass-capture geomatic technique for generating precise 3D reconstructions from a series of 2D images. Through the strategic matching of features identified from multiple overlapping 2D photographs captured from different viewing angles, a detailed and accurate 3D model of the object is produced [5,7]. This technique is implemented through a sequence of steps. Initially, key features are extracted from the images and matched across the set. This process is followed by the estimation of camera poses, the generation of a sparse point cloud, and subsequently, the application of Multi-View Stereo methods for dense point cloud creation [5]. In the feature extraction and image matching phase, several algorithms can be employed. While Scale Invariant Feature Transform (SIFT) is commonly used [4], alternative methods such as Speeded-Up Robust Features (SURF), Features from Accelerated Segment Test (FAST), and Oriented FAST and Rotated BRIEF (ORB) also offer robust solutions. These algorithms play a crucial role in identifying and matching features across the different images to ensure accurate reconstruction. Following feature matching, the SfM process involves camera pose estimation and the generation of a sparse point cloud. An essential part of this step is bundle adjustment, a process that refines the camera positions and improves the representation of the sparse point cloud by adjusting the camera parameters and 3D point positions to minimize re-projection errors [6]. This results in a more accurate and reliable 3D structure. Subsequently, the Multi-View Stereo techniques are employed, which include depth map generation and point cloud densification. These processes further enhance the detail and completeness of the 3D model by creating a dense point cloud, offering a highly detailed and comprehensive representation of the object [3]. Finally, geometric meshing and texturing are applied to this dense point cloud, culminating in the creation of the final 3D model. This sequence of steps ensures a detailed and accurate representation of the photographed object, reflecting the capabilities and adaptability of SfM-MVS photogrammetry in engineering research.

For the data collection, a Canon EOS 5DS R camera fitted with a Canon 35mm F1.4L II lens was used. The settings chosen were ISO 100 and f/11 aperture to ensure high image quality with sufficient sharpness on the entire object. Overall, a total of 122 images were captured, each image having a resolution of 8688 × 5792 pixels and an overlap of approximately 70 %. Overlap is critical to ensure sufficient data for the SfM process. During data capture, the overlap of at least 70 % was used and the beam was captured from all sides. There were four rounds of overlapping images. The first set of images was capturing the beam from a high angle looking down at an angle. The second set contains images captured around the beam with a camera pointed horizontally at the beam. The third set contains images facing up at an angle. In addition, the last set of images was captured directly below the beam, looking straight up. This allowed to capture the beam element fully with sufficient overlap. The camera network is illustrated in Fig. 4.

**Fig. 3:**
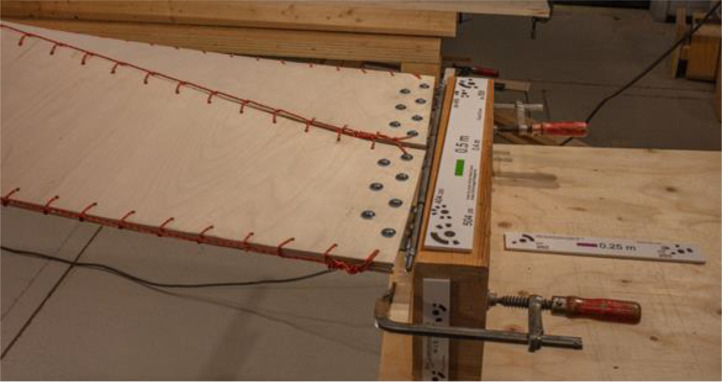
Scale bars of varying length 10–50 cm were used for scaling and accuracy control in the photogrammetric reconstruction process.

For scaling and accuracy control, six different scale bars were used for the imaging process. These scale bars, with lengths varying from 10 cm to 50 cm, served as control points and distances in the data set (see Fig. 3). To this end, they are crucial for maintaining accuracy and precision in the final 3D model. Six distances on the scale bars were employed to refine the alignment, and seven distances were used for accuracy control after the alignment.

Data processing was performed using RealityCapture software, version 1.2.1.116300 RC. The software was tasked with aligning the images using predetermined settings: high mode, 10000 maximum features per mpx and 40000 max features per image, 10000 preselector features, K + Brown4 with tangential2 lens distortion model, 1x image downscale factor, and max feature reprojection error set to 1. As a result of this process, all 122 images were aligned with a mean reprojection error of 0.391 pixels. The mean control distance error was 0.073 mm and standard deviation of 0.063 mm, which confirmed high accuracy of the photogrammetric alignment process.

**Fig. 4:**
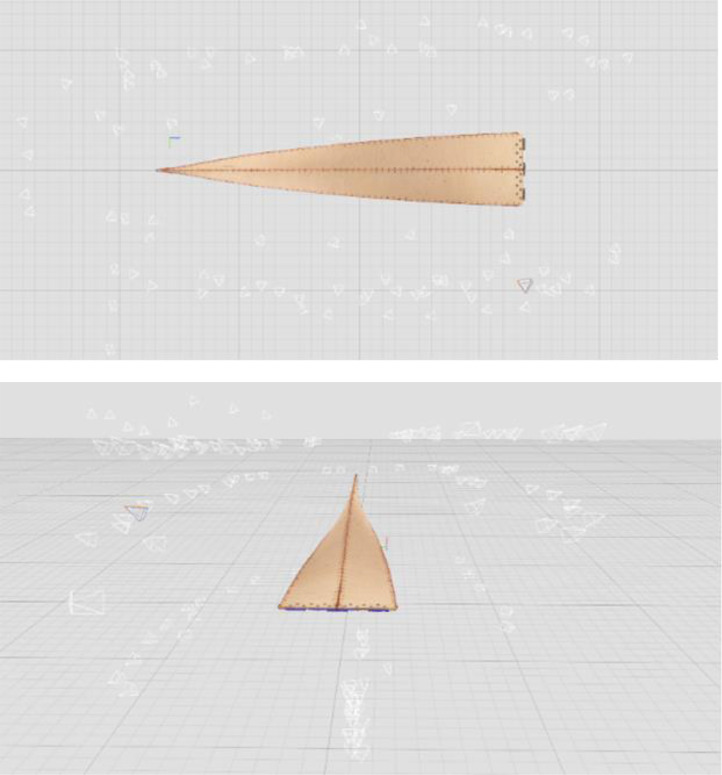
Screenshot from the photogrammetric software illustrating the reconstructed 3D model of the twisted bending-active beam element and the estimated camera network.

**Fig. 5:**
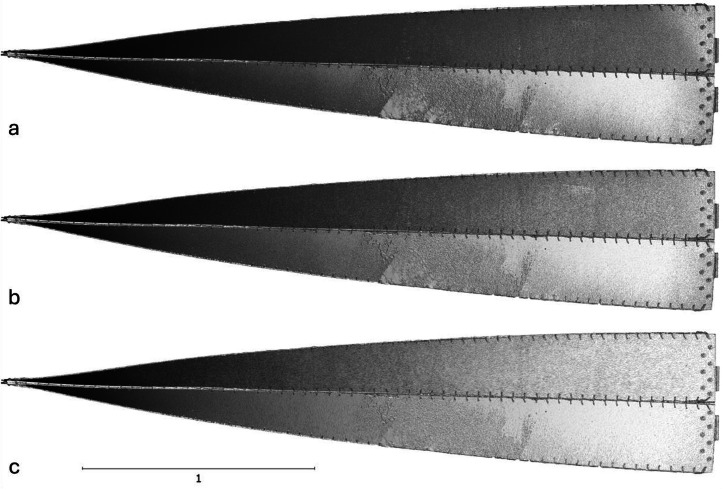
Reconstructed 3D mesh of the twisted bending-active beam element simplified to three mesh sizes: a) high-poly, b) medium-poly, and c) low poly.

**Fig. 6:**
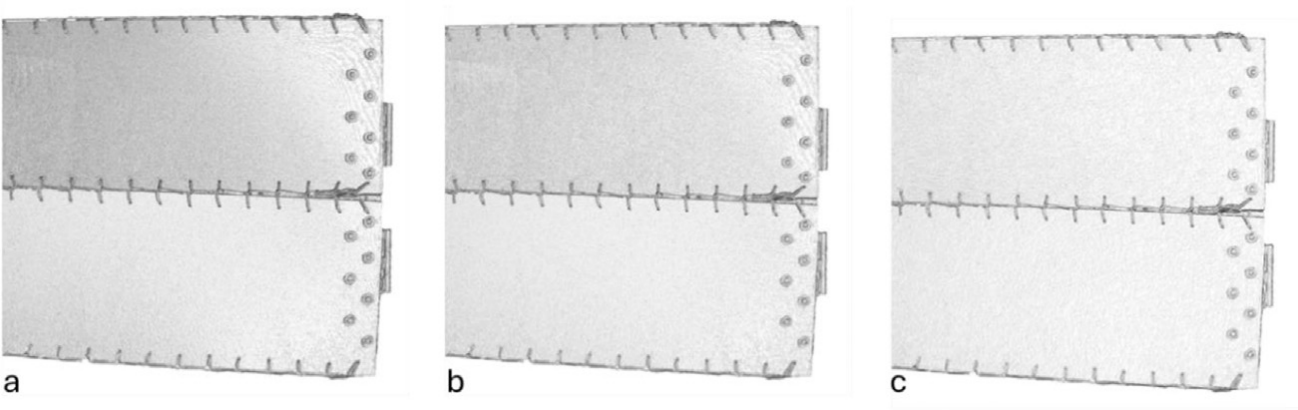
Zoomed-in view of the twisted bending-active beam element mesh with three mesh sizes: a) high-poly, b) medium-poly, and c) low poly.

The next phase involved reconstructing the data to generate a 3D model of the object. This was achieved in RealityCapture software in the Calculate model mode by setting the reconstruction mode to normal and keeping the image downscale factor at 1. The output from this stage was a high-resolution point cloud, and a raw mesh (Fig. 4). The mesh was then simplified using RealityCapture built-in mesh simplification tool to a high-poly, medium-poly, and low-poly meshes with the triangle count equal to 27.3 million, 6.8 million, and 852.4 thousand triangles, respectively. The three mesh sizes are illustrated in Figs. 5 and 6.

Finally, the high-poly mesh was textured with 6 textures of 8192 × 8192 pixels size and a texel size of 0.000109 m/texel to enhance the visual realism of the model. These textures were reprojected onto the medium- and low-poly meshes so that each simplified model had 12 textured maps (6 color maps and 6 normal maps per each model) and saved in .png format.

**Fig. 7:**
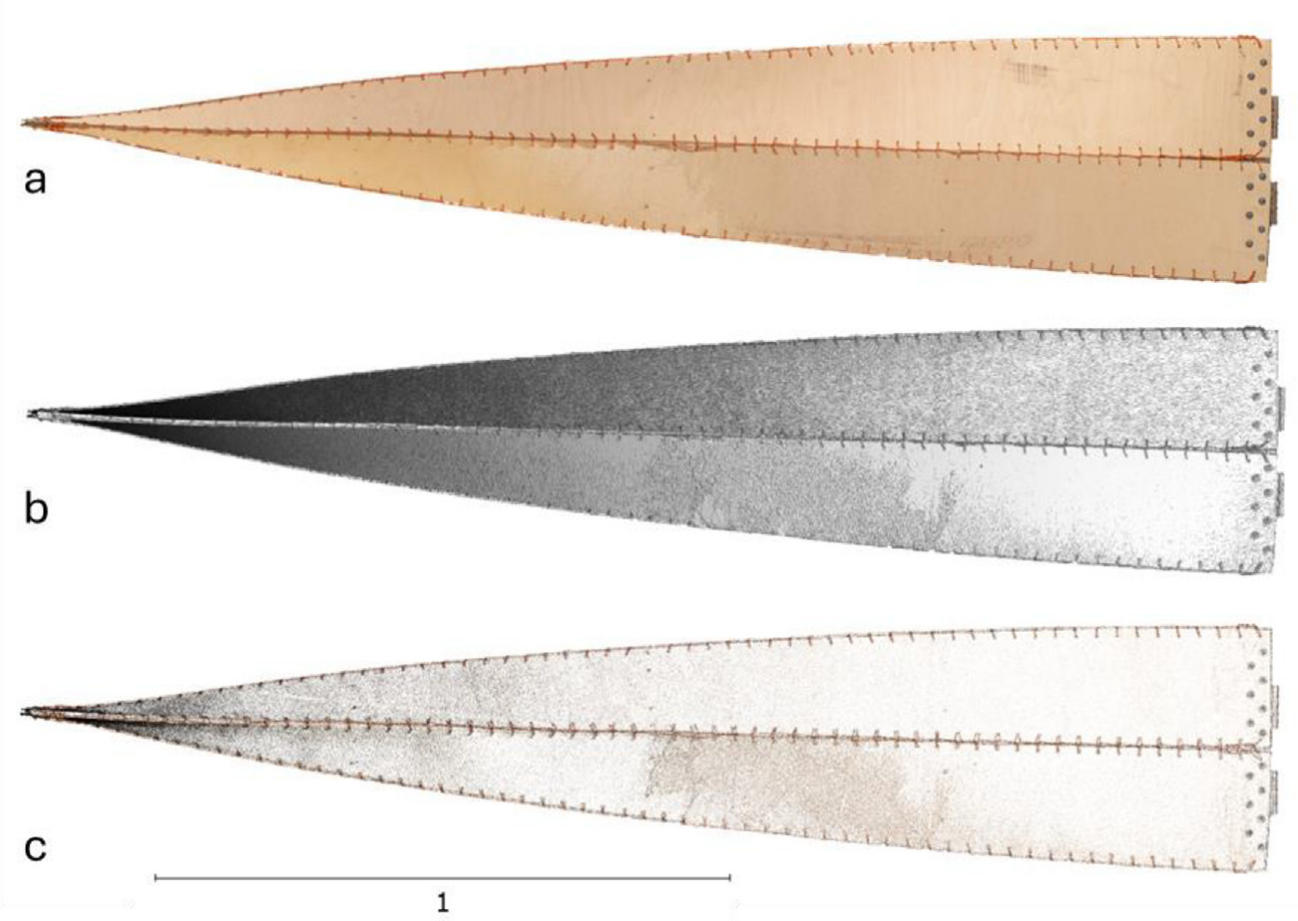
Rendered image illustrating the resulting high-poly textured mesh (a), mesh without texture (b), and the point cloud (c).

The output files from this procedure include .xyz file containing the high-resolution point cloud data, .obj files containing the mesh data in two sizes, and .png files containing the texture data. Each of these files contributes to the complete, accurate 3D reconstruction of the twisted beam element in digital environment. The final render of the textured mesh, mesh without texture, and the point cloud are given in Fig. 7.

## 5 Limitations

It is important to note that the specific algorithms utilized by the RealityCapture software, employed in our study for photogrammetric processing, are proprietary and not publicly disclosed. Therefore, while the specification of the general approach is given and examples of algorithms used in SfM and MVS are listed, the exact details of the feature extraction and image matching processes within RealityCapture remain unknown.

The reconstructed mesh of the beam element (Fig. 5a) does contain small imperfections. They are mostly present on the bottom part of the element, on the middle part of the plywood strip surface. The imperfections are less pronounced in the simplified meshes due to smoothing the mesh surface that take place when reducing the number of triangular elements on the surface. Therefore, their influence on the numerical simulations and conducted analyses is limited.

## Ethics Statement

This work did not involve human subjects, animal experiments, or data from social media platforms.

## CRediT authorship contribution statement

**Mateusz Janiszewski:** Software, Writing – original draft, Writing – review & editing. **Serenay Elmas:** Writing – original draft, Writing – review & editing. **Athanasios A. Markou:** Writing – review & editing. **Joonas Jaaranen:** Writing – review & editing. **Günther H. Filz:** Supervision.

## Declaration of Competing Interest

The authors declare that they have no known competing financial interests or personal relationships that could have appeared to influence the work reported in this paper.

## Data Availability

3D Dataset of a twisted bending-active beam element digitized using Structure-from-Motion Photogrammetry (Original data) (Mendeley Data).
